# Widespread severe acute respiratory coronavirus virus 2 (SARS-CoV-2) laboratory surveillance program to minimize asymptomatic transmission in high-risk inpatient and congregate living settings

**DOI:** 10.1017/ice.2020.301

**Published:** 2020-06-16

**Authors:** Lauren P. Jatt, Alexander Winnett, Christopher J. Graber, John Vallone, David O. Beenhouwer, Matthew Bidwell Goetz

**Affiliations:** 1 David Geffen School of Medicine at UCLA, Los Angeles, California; 2 Infectious Diseases Section, Veterans’ Affairs Greater Los Angeles Healthcare System, Los Angeles, California; 3 Pathology and Laboratory Medicine Service, Veterans’ Affairs Greater Los Angeles Healthcare System, Los Angeles, California

## Abstract

We describe a widespread laboratory surveillance program for severe acute respiratory coronavirus virus 2 (SARS-CoV-2) at an integrated medical campus that includes a tertiary-care center, a skilled nursing facility, a rehabilitation treatment center, and temporary shelter units. We identified 22 asymptomatic cases of SARS-CoV-2 and implemented infection control measures to prevent SARS-CoV-2 transmission in congregate settings.

Approximately 1.7 million cases of SARS-CoV-2 infection have been reported in the United States.^1^ In Los Angeles County, >57,000 cases and 2,443 deaths have been reported, and more than half (n = 1,233) of these deaths have occurred in residential congregate settings.^2^ Preliminary evidence shows that infection-control strategies focused solely on symptomatic individuals are insufficient to prevent transmission in congregate living facilities.^3-6^

The Veterans’ Affairs Greater Los Angeles Healthcare System (VAGLAHS) is a large healthcare facility that includes an inpatient tertiary-care center and multiple congregate living facilities on a single campus. As part of its coronavirus disease 2019 (COVID-19) response, VAGLAHS implemented a widespread laboratory surveillance program for SARS-CoV-2 in both hospital and residential facilities.

Herein, we describe the laboratory surveillance program; we discuss how data gathered influenced infection control measures; and we highlight key lessons learned during implementation.

## Methods

### Setting

VAGLAHS consists of a tertiary-care hospital with 160 acute-care beds (plus an inpatient psychiatry unit and geriatric dementia unit), a skilled nursing facility (SNF) with 3 units totaling 150 beds, and a 151-bed residential rehabilitation treatment center. Additionally, during the pandemic, VAGLAHS created temporary shelter units with capacity to shelter 218 homeless individuals.

### Laboratory testing

Either a nasopharyngeal swab in universal/viral transport media or a cobas polymerase chain reaction (PCR) media dual swab kit was used with the cobas 6800/8800 diagnostic system (Roche, Basel, Switzerland) to collect nasopharyngeal or combined nasopharyngeal and oropharyngeal specimens, respectively. Reverse-transcription (RT)-PCR was performed by a reference laboratory, the VAGLAHS Pathology and Laboratory Medicine Service (PALMS) or the Veterans’ Affairs Long Beach Healthcare System (VALBHS).

### Surveillance program planning and implementation

A daily meeting organized by facility leadership coordinated COVID-19 planning and implementation of testing. Participants included leadership from PALMS, infectious disease, nursing, inpatient medicine, outpatient medicine, SNF, the residential rehabilitation treatment center, and the temporary shelter units.

## Results

### Overview of testing availability, turnaround time, and capacity

From March 11 through 26, 2020 VAGLAHS sent all specimens for SARS-CoV-2 testing to a reference laboratory. During this time, results returned in a median of 5 days (interquartile range [IQR], 4–8). On March 27, PALMS completed their emergency use authorization for the US Food and Drug Administration and validation of in-house SARS-CoV-2 RT-PCR testing (30–60 tests per day capacity). From March 27 through 30, most RT-PCR testing was performed in house, decreasing turnaround time to a median of 2 days (IQR, 2–3). Finally, on March 31, the laboratory at VALBHS initiated SARS-CoV-2 testing using the cobas system and began accepting specimens from other VA facilities, substantially increasing testing capacity and further decreasing turnaround time to a median of 1 day (IQR, 1–1).

### Summary of all testing

**Fig. 1.**
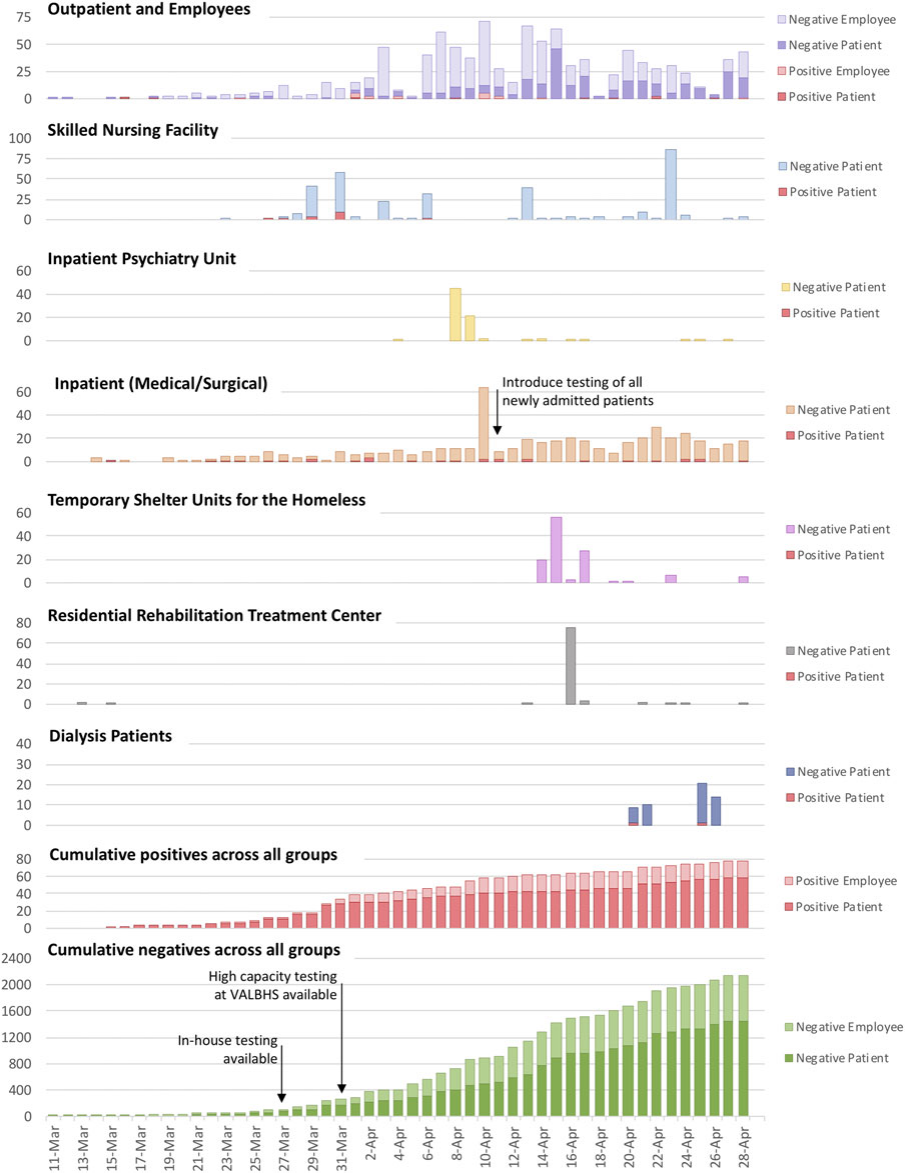
Test results of widespread laboratory surveillance program for SARS-CoV-2 in hospital and congregate living settings, March 11–April 29, 2020.

From March 11 to April 29, 2,230 tests for SARS-CoV-2 were performed on 1,781 individuals (Table 1). Of these individuals, 78 tested positive: 58 patients and 20 employees. Surveillance testing for patients was implemented over time as testing capacity increased, starting with the highest risk settings (Fig. 1). Employees were tested if they developed symptoms or had close contact with a known positive case without appropriate personal protective equipment. Upon entrance to all facilities, all individuals were asked about fever, respiratory symptoms, and/or close contact with persons known to have COVID-19; if any of these symptoms were present, the individual was appropriately triaged and was not allowed to enter the building.

**Table 1.** Characteristics of All Individuals Tested for SARS-CoV-2 Throughout the Surveillance Program by Location

Characteristic	All	Outpatient^a^	Skilled Nursing Facility	Inpatient Psychiatry	Inpatient (Medical/ Surgical)	Temporary Shelter Units for the Homeless	Residential Rehabilitation Treatment Center	Dialysis Patients
No. tested	1,781	949	149	71	353	121	82	55
Age, mean y ±SD	57.2±16.0	51.8±14.8	68.5±15.0	61.6±16.6	67.2±14.5	52.5±13.6	51.2±13.7	70.1±9.8
Male, no. (%)	1282 (72)	512 (54)	115 (77)	67 (94)	338 (96)	121 (100)	74 (90)	55 (100)
First positive SARS-CoV-2 RT-PCR, no. (%)	78 (4)	28 (3)	18 (12)	0 (0)	30 (8)	0 (0)	0 (0)	2 (4)

Note. SD, standard deviation.

a Includes 8 patients and 20 employees.

### Skilled nursing facility surveillance

On March 26 and 27, 2 symptomatic SNF residents were tested for SARS-CoV-2; on March 28, both tests returned positive. Given the high-risk implications of an outbreak and increased availability of in-house testing, all SNF residents (n = 100) were tested regardless of symptoms. This surveillance identified 14 asymptomatic SARS-CoV-2–positive residents from 2 SNF units who were moved to the tertiary-care facility and isolated under appropriate precautions. On April 3 and April 6, 57 residents who originally tested negative but lived in the same SNF unit as the SARS-CoV-2 positive individuals were retested, and 2 additional asymptomatic cases were identified. On April 13, a third round of testing was conducted among 39 residents who lived on the same unit as the SARS-CoV-2–positive residents were retested between April 3 and 6; none were positive. A fourth surveillance round (n = 87) on April 23 also yielded negative results.

### Inpatient psychiatry unit surveillance

On April 8 and 9, 45 patients from the inpatient psychiatry unit and 21 patients in the geriatric dementia unit all tested negative. Following this unit-wide testing, all new admissions were required to have a negative SARS-CoV-2 test.

### Pre-existing and new inpatient admission surveillance

On April 10, universal hospital-wide SARS-CoV-2 testing was implemented on all inpatients (medical or surgical) who had not previously been tested or tested negative on admission. Overall, 32 inpatients were tested and 2 new positive cases were identified. Also, 22 patients with a previously negative test were retested; all remained negative. From April 10 onward, all new admissions were tested and all inpatients were retested every 7 days. Between April 10 and 29, 12 new inpatient admissions tested positive, 2 of whom lacked classic COVID-19 symptoms and would otherwise not have met symptom-based testing criteria.

### Temporary shelter units for surveillance of homeless individuals

From April 14 to 17, 106 individuals participating in temporary shelter programs were tested for SARS-CoV-2; all tests were negative. Also, 2 individuals who had tested positive before arriving at the campus were housed in an area designated for SARS-CoV-2–positive patients for 14 days prior to shelter entry.

### Surveillance in the residential rehabilitation treatment center

On April 16, 76 residents from the on-campus residential rehabilitation treatment center for substance use were tested; all were negative.

### Surveillance among dialysis patients

From April 22 to 28, 55 patients receiving dialysis at VAGLAHS were tested; 2 tested positive. Although neither had symptoms or signs characteristic of COVID-19, both were admitted to acute care for isolation and monitoring.

## Discussion

To our knowledge, this is the first description of a widespread laboratory surveillance program for SARS-CoV-2 on an integrated medical campus. In the early days of the pandemic, testing capacity was limited, so efforts focused predominantly on symptomatic individuals. As testing capacity increased in early April and the importance of asymptomatic transmission was recognized, we transitioned to a more comprehensive program in which we identified 22 asymptomatic individuals who would not otherwise have been diagnosed.

Two key components enabled the success of this widespread laboratory surveillance program: (1) close collaboration with PALMS to secure access to high-volume molecular testing and (2) strong coordination of staff from multiple disciplines to implement testing.

The early initiative taken by PALMS to develop an in-house test for SARS-CoV-2 was instrumental in increasing testing capacity and decreasing turnaround time. Without this in-house test, surveillance testing of the >100 residents in the SNF within a single week would not have been possible. Similarly, the roll-out of a high-volume, high-throughput testing system at VALBHS further increased capacity to >1,900 tests over 4 weeks. Finally, to avoid shortages in specimen swabs, VAGLAHS repurposed swab kits for *Chlamydia trachomatis* and *Neisseria gonorrhoeae* testing to test for SARS-CoV-2. Close coordination and frequent communication between nurses, physicians, and other staff, facilitated by daily leadership meetings, in both the hospital and congregate living facilities were critical in implementing the surveillance program.

Overall, the implementation of a widespread surveillance testing strategy likely prevented asymptomatic transmission of SARS-CoV-2, thereby preventing potential outbreaks of COVID-19 within an integrated medical campus.

## References

[1] Cases in the US. Centers for Disease Control and Prevention website. https://www.cdc.gov/coronavirus/2019-ncov/cases-updates/cases-in-us.html Published 2020. Accessed June 3, 2020.

[2] COVID-19 in Los Angeles County. LA County Department of Public Health website. http://publichealth.lacounty.gov/media/coronavirus/locations.htm#residential-settings Published 2020. Accessed June 3, 2020.

[3] Mosites E. Assessment of SARS-CoV-2 infection prevalence in homeless shelters—four US cities, March 27–April 15, 2020. Morb Mortal Wkly Rep 2020;69:521–522. 10.15585/mmwr.mm6917e1 PMC7206983 32352957

[4] Roxby AC. Detection of SARS-CoV-2 Among residents and staff members of an independent and assisted living community for older adults—Seattle, Washington, 2020. Morb Mortal Wkly Rep 2020;69:416–418. 10.15585/mmwr.mm6914e2 PMC7147909 32271726

[5] Arons MM , Hatfield KM , Reddy SC , et al. Presymptomatic SARS-CoV-2 infections and transmission in a skilled nursing facility. N Engl J Med 2020;382:2081–2090. 32329971 10.1056/NEJMoa2008457 PMC7200056

[6] Kimball A. Asymptomatic and presymptomatic SARS-CoV-2 infections in residents of a long-term care skilled nursing facility—King County, Washington, March 2020. Morb Mortal Wkly Rep 2020;69:377–381. 10.15585/mmwr.mm6913e1 PMC7119514 32240128

